# Supplementary material for: Complete chloroplast genome assembly and phylogenetic analysis of blackcurrant (Ribes nigrum), red and white currant (Ribes rubrum), and gooseberry (Ribes uva-crispa) provide new insights into the phylogeny of Grossulariaceae
Source: PeerJ. 2023 Oct 10;11:e16272. doi: 10.7717/peerj.16272 (PMC10573389; doi:10.7717/peerj.16272)
Supplement: Supplemental Information 1 [file peerj-11-16272-s001.docx]

**Table S1.** Intron annotation of the four *Ribes* chloroplast genomes

| Species | Gene | Strand | Start | End | Exon I | Intron I | Exon II | Intron II | Exon III |
| --- | --- | --- | --- | --- | --- | --- | --- | --- | --- |
| Red/White currant  (*Ribes rubrum*) | *trnK-UUU* | - | 1,738 | 4,300 | 37 | 2,491 | 35 |  |  |
|  | *rps16* | - | 5,148 | 6,238 | 40 | 839 | 212 |  |  |
|  | *trnG-GCC* | + | 9,933 | 10,704 | 24 | 700 | 48 |  |  |
|  | *atpF* | - | 12,791 | 14,055 | 145 | 710 | 410 |  |  |
|  | *rpoC1* | - | 22,047 | 24,832 | 430 | 743 | 1,613 |  |  |
|  | *ycf3* | - | 45,090 | 47,059 | 124 | 717 | 230 | 746 | 153 |
|  | *trnL-UAA* | + | 50,303 | 50,898 | 37 | 509 | 50 |  |  |
|  | *trnV-UAC* | - | 54,533 | 55,174 | 39 | 566 | 37 |  |  |
|  | *clpP* | - | 73,286 | 75,283 | 71 | 786 | 294 | 621 | 226 |
|  | *petB* | + | 78,163 | 79,598 | 6 | 788 | 642 |  |  |
|  | *petD* | + | 79,797 | 80,958 | 8 | 673 | 481 |  |  |
|  | *rpl16* | - | 84,369 | 85,797 | 9 | 991 | 429 |  |  |
|  | *rpl2* | - | 87,501 | 88,994 | 394 | 666 | 434 |  |  |
|  | *ndhB* | - | 97,799 | 100,010 | 775 | 679 | 758 |  |  |
|  | *rps12* | + | 100,850 | 101,631 | 89 | 463 | 230 |  |  |
|  | *trnI-GAU* | + | 105,555 | 106,403 | 42 | 772 | 35 |  |  |
|  | *trnA-UGC* | + | 106,468 | 107,373 | 38 | 826 | 42 |  |  |
|  | *ndhA* | - | 123,239 | 125,340 | 553 | 1,010 | 539 |  |  |
|  | *trnA-UGC* | - | 137,799 | 138,704 | 38 | 826 | 42 |  |  |
|  | *trnI-GAU* | - | 138,769 | 139,617 | 42 | 772 | 35 |  |  |
|  | *rps12* | - | 143,541 | 144,322 | 230 | 463 | 89 |  |  |
|  | *ndhB* | + | 145,162 | 147,373 | 775 | 679 | 758 |  |  |
|  | *rpl2* | + | 156,178 | 157,671 | 394 | 666 | 434 |  |  |
| Blackcurrant  (*Ribes nigrum*) | *trnK-UUU* | - | 1,747 | 4,302 | 37 | 2,484 | 35 |  |  |
|  | *rps16* | - | 5,131 | 6,222 | 40 | 840 | 212 |  |  |
|  | *trnG-GCC* | + | 9,684 | 10,457 | 24 | 702 | 48 |  |  |
|  | *atpF* | - | 12,528 | 13,792 | 145 | 710 | 410 |  |  |
|  | *rpoC1* | - | 21,804 | 24,578 | 430 | 732 | 1,613 |  |  |
|  | *ycf3* | - | 44,706 | 46,674 | 124 | 717 | 230 | 745 | 153 |
|  | *trnL-UAA* | + | 49,932 | 50,533 | 37 | 515 | 50 |  |  |
|  | *trnV-UAC* | - | 54,174 | 54,818 | 39 | 569 | 37 |  |  |
|  | *clpP* | - | 72,957 | 74,956 | 71 | 788 | 294 | 621 | 226 |
|  | *petB* | + | 77,841 | 79,291 | 6 | 803 | 642 |  |  |
|  | *petD* | + | 79,490 | 80,657 | 8 | 679 | 481 |  |  |
|  | *rpl16* | - | 84,072 | 85,504 | 9 | 995 | 429 |  |  |
|  | *rpl2* | - | 87,210 | 88,704 | 394 | 667 | 434 |  |  |
|  | *ndhB* | - | 97,502 | 99,713 | 775 | 679 | 758 |  |  |
|  | *rps12* | + | 100,553 | 101,334 | 89 | 463 | 230 |  |  |
|  | *trnI-GAU* | + | 105,258 | 106,108 | 42 | 774 | 35 |  |  |
|  | *trnA-UGC* | + | 106,173 | 107,078 | 38 | 826 | 42 |  |  |
|  | *ndhA* | - | 123,003 | 125,104 | 553 | 1,010 | 539 |  |  |
|  | *trnA-UGC* | - | 137,459 | 138,364 | 38 | 826 | 42 |  |  |
|  | *trnI-GAU* | - | 138,429 | 139,279 | 42 | 774 | 35 |  |  |
|  | *rps12* | - | 143,203 | 143,984 | 230 | 463 | 89 |  |  |
|  | *ndhB* | + | 144,824 | 147,035 | 775 | 679 | 758 |  |  |
|  | *rpl2* | + | 155,833 | 157,327 | 394 | 667 | 434 |  |  |
| Gooseberry  (*Ribes uva-crispa*) | *trnK-UUU* | - | 1,760 | 4,316 | 37 | 2,485 | 35 |  |  |
|  | *rps16* | - | 5,198 | 6,288 | 40 | 839 | 212 |  |  |
|  | *trnG-GCC* | + | 10,090 | 10,860 | 24 | 699 | 48 |  |  |
|  | *atpF* | - | 12,931 | 14,195 | 145 | 710 | 410 |  |  |
|  | *rpoC1* | - | 22,198 | 24,972 | 430 | 732 | 1,613 |  |  |
|  | *ycf3* | - | 45,079 | 47,044 | 124 | 717 | 230 | 742 | 153 |
|  | *trnL-UAA* | + | 50,267 | 50,868 | 37 | 515 | 50 |  |  |
|  | *trnV-UAC* | - | 54,521 | 55,160 | 39 | 564 | 37 |  |  |
|  | *clpP* | - | 73,269 | 75,268 | 71 | 788 | 294 | 621 | 226 |
|  | *petB* | + | 78,153 | 79,589 | 6 | 789 | 642 |  |  |
|  | *petD* | + | 79,788 | 80,955 | 8 | 679 | 481 |  |  |
|  | *rpl16* | - | 84,355 | 85,787 | 9 | 995 | 429 |  |  |
|  | *rpl2* | - | 87,492 | 88,986 | 394 | 667 | 434 |  |  |
|  | *ndhB* | - | 97,790 | 100,001 | 775 | 679 | 758 |  |  |
|  | *rps12* | + | 100,841 | 101,622 | 89 | 463 | 230 |  |  |
|  | *trnI-GAU* | + | 105,546 | 106,396 | 42 | 774 | 35 |  |  |
|  | *trnA-UGC* | + | 106,461 | 107,366 | 38 | 826 | 42 |  |  |
|  | *ndhA* | - | 123,112 | 125,194 | 553 | 991 | 539 |  |  |
|  | *trnA-UGC* | - | 137,663 | 138,568 | 38 | 826 | 42 |  |  |
|  | *trnI-GAU* | - | 138,633 | 139,483 | 42 | 774 | 35 |  |  |
|  | *rps12* | - | 143,407 | 144,188 | 230 | 463 | 89 |  |  |
|  | *ndhB* | + | 145,028 | 147,239 | 775 | 679 | 758 |  |  |
|  | *rpl2* | + | 156,043 | 157,537 | 394 | 667 | 434 |  |  |

**Table S2.** Tandem repeats in four *Ribes* taxa

| **Species** | **Indices** | **Period Size** | **Copy Number** | **Consensus Size** | **Percent Matches** | **Entropy (0-2)** |
| --- | --- | --- | --- | --- | --- | --- |
| Red/White currant  (*Ribes rubrum*) | 4,731—4,755 | 13 | 1.9 | 13 | 100 | 1.74 |
|  | 5,038—5,086 | 17 | 2.8 | 17 | 90 | 1.26 |
|  | 9,095—9,119 | 12 | 2.1 | 12 | 100 | 1.36 |
|  | 30,553—30,586 | 17 | 2.0 | 17 | 88 | 1.74 |
|  | 39,202—39,238 | 14 | 2.6 | 15 | 87 | 1.74 |
|  | 62,337—62,381 | 17 | 2.8 | 16 | 80 | 1.59 |
|  | 82,581—82,626 | 24 | 1.9 | 24 | 86 | 1.95 |
|  | 84,895—84,932 | 17 | 2.2 | 17 | 90 | 1.73 |
|  | 92,216—92,304 | 21 | 4.1 | 21 | 81 | 1.68 |
|  | 94,262—94,291 | 15 | 2.0 | 15 | 100 | 1.80 |
|  | 94,670—94,741 | 18 | 4.2 | 18 | 91 | 1.82 |
|  | 102,550—102,584 | 14 | 2.4 | 14 | 90 | 1.63 |
|  | 107,008—107,082 | 38 | 2.0 | 37 | 97 | 1.97 |
|  | 110,598—110,663 | 32 | 2.1 | 32 | 97 | 1.84 |
|  | 127,658—127,691 | 15 | 2.2 | 16 | 89 | 1.33 |
|  | 134,509—134,574 | 32 | 2.1 | 32 | 97 | 1.84 |
|  | 138,090—138,164 | 38 | 2.0 | 37 | 97 | 1.97 |
|  | 142,588—142,622 | 14 | 2.4 | 14 | 90 | 1.63 |
|  | 150,431—150,490 | 18 | 3.3 | 18 | 95 | 1.86 |
|  | 150,431—150,489 | 9 | 6.6 | 9 | 72 | 1.83 |
|  | 150,438—150,502 | 18 | 3.8 | 17 | 92 | 1.84 |
|  | 150,881—150,910 | 15 | 2.0 | 15 | 100 | 1.80 |
|  | 152,868—152,956 | 21 | 4.1 | 21 | 81 | 1.68 |
| Blackcurrant  (*Ribes nigrum*) | [4,731—4,755](file:///D:\\%25E6%25A1%258C%25E9%259D%25A2\\%25E5%258F%25B6%25E7%25BB%25BF%25E4%25BD%2593%25E5%259F%25BA%25E5%259B%25A0%25E7%25BB%2584%25E6%2595%25B0%25E6%258D%25AE\\%25E7%25BB%2584%25E8%25A3%2585%25E7%259A%2584%25E5%258F%25B6%25E7%25BB%25BF%25E4%25BD%2593%25E4%25BF%25A1%25E6%2581%25AF\\RN\\CPGAVAS2\\166073344774101.fas.2.7.7.80.10.50.500.1.txt.html#4731--4755,13,1.9,13,1) | 13 | 1.9 | 13 | 100 | 1.74 |
|  | [38,216—38,245](file:///D:\\%25E6%25A1%258C%25E9%259D%25A2\\%25E5%258F%25B6%25E7%25BB%25BF%25E4%25BD%2593%25E5%259F%25BA%25E5%259B%25A0%25E7%25BB%2584%25E6%2595%25B0%25E6%258D%25AE\\%25E7%25BB%2584%25E8%25A3%2585%25E7%259A%2584%25E5%258F%25B6%25E7%25BB%25BF%25E4%25BD%2593%25E4%25BF%25A1%25E6%2581%25AF\\RN\\CPGAVAS2\\166073344774101.fas.2.7.7.80.10.50.500.1.txt.html#38216--38245,15,2.0,15,2) | 15 | 2.0 | 15 | 93 | 1.77 |
|  | [49,371—49,397](file:///D:\\%25E6%25A1%258C%25E9%259D%25A2\\%25E5%258F%25B6%25E7%25BB%25BF%25E4%25BD%2593%25E5%259F%25BA%25E5%259B%25A0%25E7%25BB%2584%25E6%2595%25B0%25E6%258D%25AE\\%25E7%25BB%2584%25E8%25A3%2585%25E7%259A%2584%25E5%258F%25B6%25E7%25BB%25BF%25E4%25BD%2593%25E4%25BF%25A1%25E6%2581%25AF\\RN\\CPGAVAS2\\166073344774101.fas.2.7.7.80.10.50.500.1.txt.html#49371--49397,12,2.2,12,3) | 12 | 2.2 | 12 | 100 | 1.60 |
|  | [62,007—62,051](file:///D:\\%25E6%25A1%258C%25E9%259D%25A2\\%25E5%258F%25B6%25E7%25BB%25BF%25E4%25BD%2593%25E5%259F%25BA%25E5%259B%25A0%25E7%25BB%2584%25E6%2595%25B0%25E6%258D%25AE\\%25E7%25BB%2584%25E8%25A3%2585%25E7%259A%2584%25E5%258F%25B6%25E7%25BB%25BF%25E4%25BD%2593%25E4%25BF%25A1%25E6%2581%25AF\\RN\\CPGAVAS2\\166073344774101.fas.2.7.7.80.10.50.500.1.txt.html#62007--62051,17,2.8,16,5) | 17 | 2.8 | 16 | 80 | 1.59 |
|  | [82,283—82,328](file:///D:\\%25E6%25A1%258C%25E9%259D%25A2\\%25E5%258F%25B6%25E7%25BB%25BF%25E4%25BD%2593%25E5%259F%25BA%25E5%259B%25A0%25E7%25BB%2584%25E6%2595%25B0%25E6%258D%25AE\\%25E7%25BB%2584%25E8%25A3%2585%25E7%259A%2584%25E5%258F%25B6%25E7%25BB%25BF%25E4%25BD%2593%25E4%25BF%25A1%25E6%2581%25AF\\RN\\CPGAVAS2\\166073344774101.fas.2.7.7.80.10.50.500.1.txt.html#82283--82328,24,1.9,24,6) | 24 | 1.9 | 24 | 86 | 1.95 |
|  | [84,603—84,640](file:///D:\\%25E6%25A1%258C%25E9%259D%25A2\\%25E5%258F%25B6%25E7%25BB%25BF%25E4%25BD%2593%25E5%259F%25BA%25E5%259B%25A0%25E7%25BB%2584%25E6%2595%25B0%25E6%258D%25AE\\%25E7%25BB%2584%25E8%25A3%2585%25E7%259A%2584%25E5%258F%25B6%25E7%25BB%25BF%25E4%25BD%2593%25E4%25BF%25A1%25E6%2581%25AF\\RN\\CPGAVAS2\\166073344774101.fas.2.7.7.80.10.50.500.1.txt.html#84603--84640,17,2.2,17,7) | 17 | 2.2 | 17 | 90 | 1.73 |
|  | [91,946—91,997](file:///D:\\%25E6%25A1%258C%25E9%259D%25A2\\%25E5%258F%25B6%25E7%25BB%25BF%25E4%25BD%2593%25E5%259F%25BA%25E5%259B%25A0%25E7%25BB%2584%25E6%2595%25B0%25E6%258D%25AE\\%25E7%25BB%2584%25E8%25A3%2585%25E7%259A%2584%25E5%258F%25B6%25E7%25BB%25BF%25E4%25BD%2593%25E4%25BF%25A1%25E6%2581%25AF\\RN\\CPGAVAS2\\166073344774101.fas.2.7.7.80.10.50.500.1.txt.html#91946--91997,21,2.5,21,8) | 21 | 2.5 | 21 | 100 | 1.63 |
|  | [91,926—92,014](file:///D:\\%25E6%25A1%258C%25E9%259D%25A2\\%25E5%258F%25B6%25E7%25BB%25BF%25E4%25BD%2593%25E5%259F%25BA%25E5%259B%25A0%25E7%25BB%2584%25E6%2595%25B0%25E6%258D%25AE\\%25E7%25BB%2584%25E8%25A3%2585%25E7%259A%2584%25E5%258F%25B6%25E7%25BB%25BF%25E4%25BD%2593%25E4%25BF%25A1%25E6%2581%25AF\\RN\\CPGAVAS2\\166073344774101.fas.2.7.7.80.10.50.500.1.txt.html#91926--92014,21,4.1,21,9) | 21 | 4.1 | 21 | 84 | 1.68 |
|  | [93,972—94,001](file:///D:\\%25E6%25A1%258C%25E9%259D%25A2\\%25E5%258F%25B6%25E7%25BB%25BF%25E4%25BD%2593%25E5%259F%25BA%25E5%259B%25A0%25E7%25BB%2584%25E6%2595%25B0%25E6%258D%25AE\\%25E7%25BB%2584%25E8%25A3%2585%25E7%259A%2584%25E5%258F%25B6%25E7%25BB%25BF%25E4%25BD%2593%25E4%25BF%25A1%25E6%2581%25AF\\RN\\CPGAVAS2\\166073344774101.fas.2.7.7.80.10.50.500.1.txt.html#93972--94001,15,2.0,15,10) | 15 | 2.0 | 15 | 100 | 1.80 |
|  | [94,380—94,451](file:///D:\\%25E6%25A1%258C%25E9%259D%25A2\\%25E5%258F%25B6%25E7%25BB%25BF%25E4%25BD%2593%25E5%259F%25BA%25E5%259B%25A0%25E7%25BB%2584%25E6%2595%25B0%25E6%258D%25AE\\%25E7%25BB%2584%25E8%25A3%2585%25E7%259A%2584%25E5%258F%25B6%25E7%25BB%25BF%25E4%25BD%2593%25E4%25BF%25A1%25E6%2581%25AF\\RN\\CPGAVAS2\\166073344774101.fas.2.7.7.80.10.50.500.1.txt.html#94380--94451,18,4.2,18,12) | 18 | 4.2 | 18 | 91 | 1.82 |
|  | [102,247—102,281](file:///D:\\%25E6%25A1%258C%25E9%259D%25A2\\%25E5%258F%25B6%25E7%25BB%25BF%25E4%25BD%2593%25E5%259F%25BA%25E5%259B%25A0%25E7%25BB%2584%25E6%2595%25B0%25E6%258D%25AE\\%25E7%25BB%2584%25E8%25A3%2585%25E7%259A%2584%25E5%258F%25B6%25E7%25BB%25BF%25E4%25BD%2593%25E4%25BF%25A1%25E6%2581%25AF\\RN\\CPGAVAS2\\166073344774101.fas.2.7.7.80.10.50.500.1.txt.html#102247--102281,14,2.4,14,13) | 14 | 2.4 | 14 | 90 | 1.63 |
|  | [106,713—106,787](file:///D:\\%25E6%25A1%258C%25E9%259D%25A2\\%25E5%258F%25B6%25E7%25BB%25BF%25E4%25BD%2593%25E5%259F%25BA%25E5%259B%25A0%25E7%25BB%2584%25E6%2595%25B0%25E6%258D%25AE\\%25E7%25BB%2584%25E8%25A3%2585%25E7%259A%2584%25E5%258F%25B6%25E7%25BB%25BF%25E4%25BD%2593%25E4%25BF%25A1%25E6%2581%25AF\\RN\\CPGAVAS2\\166073344774101.fas.2.7.7.80.10.50.500.1.txt.html#106713--106787,38,2.0,37,14) | 38 | 2.0 | 37 | 97 | 1.97 |
|  | [110,303—110,368](file:///D:\\%25E6%25A1%258C%25E9%259D%25A2\\%25E5%258F%25B6%25E7%25BB%25BF%25E4%25BD%2593%25E5%259F%25BA%25E5%259B%25A0%25E7%25BB%2584%25E6%2595%25B0%25E6%258D%25AE\\%25E7%25BB%2584%25E8%25A3%2585%25E7%259A%2584%25E5%258F%25B6%25E7%25BB%25BF%25E4%25BD%2593%25E4%25BF%25A1%25E6%2581%25AF\\RN\\CPGAVAS2\\166073344774101.fas.2.7.7.80.10.50.500.1.txt.html#110303--110368,32,2.1,32,15) | 32 | 2.1 | 32 | 97 | 1.84 |
|  | [127,425—127,458](file:///D:\\%25E6%25A1%258C%25E9%259D%25A2\\%25E5%258F%25B6%25E7%25BB%25BF%25E4%25BD%2593%25E5%259F%25BA%25E5%259B%25A0%25E7%25BB%2584%25E6%2595%25B0%25E6%258D%25AE\\%25E7%25BB%2584%25E8%25A3%2585%25E7%259A%2584%25E5%258F%25B6%25E7%25BB%25BF%25E4%25BD%2593%25E4%25BF%25A1%25E6%2581%25AF\\RN\\CPGAVAS2\\166073344774101.fas.2.7.7.80.10.50.500.1.txt.html#127425--127458,15,2.2,16,16) | 15 | 2.2 | 16 | 89 | 1.31 |
|  | [134,169—134,234](file:///D:\\%25E6%25A1%258C%25E9%259D%25A2\\%25E5%258F%25B6%25E7%25BB%25BF%25E4%25BD%2593%25E5%259F%25BA%25E5%259B%25A0%25E7%25BB%2584%25E6%2595%25B0%25E6%258D%25AE\\%25E7%25BB%2584%25E8%25A3%2585%25E7%259A%2584%25E5%258F%25B6%25E7%25BB%25BF%25E4%25BD%2593%25E4%25BF%25A1%25E6%2581%25AF\\RN\\CPGAVAS2\\166073344774101.fas.2.7.7.80.10.50.500.1.txt.html#134169--134234,32,2.1,32,18) | 32 | 2.1 | 32 | 97 | 1.84 |
|  | [137,750—137,824](file:///D:\\%25E6%25A1%258C%25E9%259D%25A2\\%25E5%258F%25B6%25E7%25BB%25BF%25E4%25BD%2593%25E5%259F%25BA%25E5%259B%25A0%25E7%25BB%2584%25E6%2595%25B0%25E6%258D%25AE\\%25E7%25BB%2584%25E8%25A3%2585%25E7%259A%2584%25E5%258F%25B6%25E7%25BB%25BF%25E4%25BD%2593%25E4%25BF%25A1%25E6%2581%25AF\\RN\\CPGAVAS2\\166073344774101.fas.2.7.7.80.10.50.500.1.txt.html#137750--137824,38,2.0,37,19) | 38 | 2.0 | 37 | 97 | 1.97 |
|  | [142,256—142,290](file:///D:\\%25E6%25A1%258C%25E9%259D%25A2\\%25E5%258F%25B6%25E7%25BB%25BF%25E4%25BD%2593%25E5%259F%25BA%25E5%259B%25A0%25E7%25BB%2584%25E6%2595%25B0%25E6%258D%25AE\\%25E7%25BB%2584%25E8%25A3%2585%25E7%259A%2584%25E5%258F%25B6%25E7%25BB%25BF%25E4%25BD%2593%25E4%25BF%25A1%25E6%2581%25AF\\RN\\CPGAVAS2\\166073344774101.fas.2.7.7.80.10.50.500.1.txt.html#142256--142290,14,2.4,14,20) | 14 | 2.4 | 14 | 90 | 1.63 |
|  | [150,086—150,145](file:///D:\\%25E6%25A1%258C%25E9%259D%25A2\\%25E5%258F%25B6%25E7%25BB%25BF%25E4%25BD%2593%25E5%259F%25BA%25E5%259B%25A0%25E7%25BB%2584%25E6%2595%25B0%25E6%258D%25AE\\%25E7%25BB%2584%25E8%25A3%2585%25E7%259A%2584%25E5%258F%25B6%25E7%25BB%25BF%25E4%25BD%2593%25E4%25BF%25A1%25E6%2581%25AF\\RN\\CPGAVAS2\\166073344774101.fas.2.7.7.80.10.50.500.1.txt.html#150086--150145,18,3.3,18,21) | 18 | 3.3 | 18 | 95 | 1.86 |
|  | [150,086—150,144](file:///D:\\%25E6%25A1%258C%25E9%259D%25A2\\%25E5%258F%25B6%25E7%25BB%25BF%25E4%25BD%2593%25E5%259F%25BA%25E5%259B%25A0%25E7%25BB%2584%25E6%2595%25B0%25E6%258D%25AE\\%25E7%25BB%2584%25E8%25A3%2585%25E7%259A%2584%25E5%258F%25B6%25E7%25BB%25BF%25E4%25BD%2593%25E4%25BF%25A1%25E6%2581%25AF\\RN\\CPGAVAS2\\166073344774101.fas.2.7.7.80.10.50.500.1.txt.html#150086--150144,9,6.6,9,22) | 9 | 6.6 | 9 | 72 | 1.83 |
|  | [150,093—150,157](file:///D:\\%25E6%25A1%258C%25E9%259D%25A2\\%25E5%258F%25B6%25E7%25BB%25BF%25E4%25BD%2593%25E5%259F%25BA%25E5%259B%25A0%25E7%25BB%2584%25E6%2595%25B0%25E6%258D%25AE\\%25E7%25BB%2584%25E8%25A3%2585%25E7%259A%2584%25E5%258F%25B6%25E7%25BB%25BF%25E4%25BD%2593%25E4%25BF%25A1%25E6%2581%25AF\\RN\\CPGAVAS2\\166073344774101.fas.2.7.7.80.10.50.500.1.txt.html#150093--150157,18,3.8,17,23) | 18 | 3.8 | 17 | 92 | 1.84 |
|  | [150,536—150,565](file:///D:\\%25E6%25A1%258C%25E9%259D%25A2\\%25E5%258F%25B6%25E7%25BB%25BF%25E4%25BD%2593%25E5%259F%25BA%25E5%259B%25A0%25E7%25BB%2584%25E6%2595%25B0%25E6%258D%25AE\\%25E7%25BB%2584%25E8%25A3%2585%25E7%259A%2584%25E5%258F%25B6%25E7%25BB%25BF%25E4%25BD%2593%25E4%25BF%25A1%25E6%2581%25AF\\RN\\CPGAVAS2\\166073344774101.fas.2.7.7.80.10.50.500.1.txt.html#150536--150565,15,2.0,15,24) | 15 | 2.0 | 15 | 100 | 1.80 |
|  | [152,540—152,591](file:///D:\\%25E6%25A1%258C%25E9%259D%25A2\\%25E5%258F%25B6%25E7%25BB%25BF%25E4%25BD%2593%25E5%259F%25BA%25E5%259B%25A0%25E7%25BB%2584%25E6%2595%25B0%25E6%258D%25AE\\%25E7%25BB%2584%25E8%25A3%2585%25E7%259A%2584%25E5%258F%25B6%25E7%25BB%25BF%25E4%25BD%2593%25E4%25BF%25A1%25E6%2581%25AF\\RN\\CPGAVAS2\\166073344774101.fas.2.7.7.80.10.50.500.1.txt.html#152540--152591,21,2.5,21,26) | 21 | 2.5 | 21 | 100 | 1.63 |
| Gooseberry  (*Ribes uva-crispa*) | [4,732—4,756](file:///D:\\%25E6%25A1%258C%25E9%259D%25A2\\%25E5%258F%25B6%25E7%25BB%25BF%25E4%25BD%2593%25E5%259F%25BA%25E5%259B%25A0%25E7%25BB%2584%25E6%2595%25B0%25E6%258D%25AE\\%25E7%25BB%2584%25E8%25A3%2585%25E7%259A%2584%25E5%258F%25B6%25E7%25BB%25BF%25E4%25BD%2593%25E4%25BF%25A1%25E6%2581%25AF\\CL\\CPGAVAS2\\166073366105132.fas.2.7.7.80.10.50.500.1.txt.html#4732--4756,13,1.9,13,1) | 13 | 1.9 | 13 | 100 | 1.74 |
|  | [5,028—5,083](file:///D:\\%25E6%25A1%258C%25E9%259D%25A2\\%25E5%258F%25B6%25E7%25BB%25BF%25E4%25BD%2593%25E5%259F%25BA%25E5%259B%25A0%25E7%25BB%2584%25E6%2595%25B0%25E6%258D%25AE\\%25E7%25BB%2584%25E8%25A3%2585%25E7%259A%2584%25E5%258F%25B6%25E7%25BB%25BF%25E4%25BD%2593%25E4%25BF%25A1%25E6%2581%25AF\\CL\\CPGAVAS2\\166073366105132.fas.2.7.7.80.10.50.500.1.txt.html#5028--5083,29,1.9,29,2) | 29 | 1.9 | 29 | 100 | 1.44 |
|  | [5,063—5,109](file:///D:\\%25E6%25A1%258C%25E9%259D%25A2\\%25E5%258F%25B6%25E7%25BB%25BF%25E4%25BD%2593%25E5%259F%25BA%25E5%259B%25A0%25E7%25BB%2584%25E6%2595%25B0%25E6%258D%25AE\\%25E7%25BB%2584%25E8%25A3%2585%25E7%259A%2584%25E5%258F%25B6%25E7%25BB%25BF%25E4%25BD%2593%25E4%25BF%25A1%25E6%2581%25AF\\CL\\CPGAVAS2\\166073366105132.fas.2.7.7.80.10.50.500.1.txt.html#5063--5109,20,2.4,20,3) | 20 | 2.3 | 20 | 96 | 1.34 |
|  | [5,102—5,137](file:///D:\\%25E6%25A1%258C%25E9%259D%25A2\\%25E5%258F%25B6%25E7%25BB%25BF%25E4%25BD%2593%25E5%259F%25BA%25E5%259B%25A0%25E7%25BB%2584%25E6%2595%25B0%25E6%258D%25AE\\%25E7%25BB%2584%25E8%25A3%2585%25E7%259A%2584%25E5%258F%25B6%25E7%25BB%25BF%25E4%25BD%2593%25E4%25BF%25A1%25E6%2581%25AF\\CL\\CPGAVAS2\\166073366105132.fas.2.7.7.80.10.50.500.1.txt.html#5102--5137,16,2.2,16,4) | 16 | 2.2 | 16 | 100 | 1.27 |
|  | [33,566—33,599](file:///D:\\%25E6%25A1%258C%25E9%259D%25A2\\%25E5%258F%25B6%25E7%25BB%25BF%25E4%25BD%2593%25E5%259F%25BA%25E5%259B%25A0%25E7%25BB%2584%25E6%2595%25B0%25E6%258D%25AE\\%25E7%25BB%2584%25E8%25A3%2585%25E7%259A%2584%25E5%258F%25B6%25E7%25BB%25BF%25E4%25BD%2593%25E4%25BF%25A1%25E6%2581%25AF\\CL\\CPGAVAS2\\166073366105132.fas.2.7.7.80.10.50.500.1.txt.html#33566--33599,17,2.0,17,5) | 17 | 2.0 | 17 | 94 | 1.55 |
|  | [34,561—34,588](file:///D:\\%25E6%25A1%258C%25E9%259D%25A2\\%25E5%258F%25B6%25E7%25BB%25BF%25E4%25BD%2593%25E5%259F%25BA%25E5%259B%25A0%25E7%25BB%2584%25E6%2595%25B0%25E6%258D%25AE\\%25E7%25BB%2584%25E8%25A3%2585%25E7%259A%2584%25E5%258F%25B6%25E7%25BB%25BF%25E4%25BD%2593%25E4%25BF%25A1%25E6%2581%25AF\\CL\\CPGAVAS2\\166073366105132.fas.2.7.7.80.10.50.500.1.txt.html#34561--34588,12,2.3,12,6) | 12 | 2.3 | 12 | 100 | 1.78 |
|  | [38,593—38,622](file:///D:\\%25E6%25A1%258C%25E9%259D%25A2\\%25E5%258F%25B6%25E7%25BB%25BF%25E4%25BD%2593%25E5%259F%25BA%25E5%259B%25A0%25E7%25BB%2584%25E6%2595%25B0%25E6%258D%25AE\\%25E7%25BB%2584%25E8%25A3%2585%25E7%259A%2584%25E5%258F%25B6%25E7%25BB%25BF%25E4%25BD%2593%25E4%25BF%25A1%25E6%2581%25AF\\CL\\CPGAVAS2\\166073366105132.fas.2.7.7.80.10.50.500.1.txt.html#38593--38622,15,2.0,15,7) | 15 | 2.0 | 15 | 93 | 1.77 |
|  | [38,852—38,885](file:///D:\\%25E6%25A1%258C%25E9%259D%25A2\\%25E5%258F%25B6%25E7%25BB%25BF%25E4%25BD%2593%25E5%259F%25BA%25E5%259B%25A0%25E7%25BB%2584%25E6%2595%25B0%25E6%258D%25AE\\%25E7%25BB%2584%25E8%25A3%2585%25E7%259A%2584%25E5%258F%25B6%25E7%25BB%25BF%25E4%25BD%2593%25E4%25BF%25A1%25E6%2581%25AF\\CL\\CPGAVAS2\\166073366105132.fas.2.7.7.80.10.50.500.1.txt.html#38852--38885,14,2.4,14,8) | 14 | 2.4 | 14 | 95 | 1.38 |
|  | [49,614—49,639](file:///D:\\%25E6%25A1%258C%25E9%259D%25A2\\%25E5%258F%25B6%25E7%25BB%25BF%25E4%25BD%2593%25E5%259F%25BA%25E5%259B%25A0%25E7%25BB%2584%25E6%2595%25B0%25E6%258D%25AE\\%25E7%25BB%2584%25E8%25A3%2585%25E7%259A%2584%25E5%258F%25B6%25E7%25BB%25BF%25E4%25BD%2593%25E4%25BF%25A1%25E6%2581%25AF\\CL\\CPGAVAS2\\166073366105132.fas.2.7.7.80.10.50.500.1.txt.html#49614--49639,12,2.2,12,9) | 12 | 2.2 | 12 | 100 | 1.54 |
|  | [62,333—62,377](file:///D:\\%25E6%25A1%258C%25E9%259D%25A2\\%25E5%258F%25B6%25E7%25BB%25BF%25E4%25BD%2593%25E5%259F%25BA%25E5%259B%25A0%25E7%25BB%2584%25E6%2595%25B0%25E6%258D%25AE\\%25E7%25BB%2584%25E8%25A3%2585%25E7%259A%2584%25E5%258F%25B6%25E7%25BB%25BF%25E4%25BD%2593%25E4%25BF%25A1%25E6%2581%25AF\\CL\\CPGAVAS2\\166073366105132.fas.2.7.7.80.10.50.500.1.txt.html#62333--62377,17,2.8,16,11) | 17 | 2.8 | 16 | 80 | 1.59 |
|  | [82,578—82,623](file:///D:\\%25E6%25A1%258C%25E9%259D%25A2\\%25E5%258F%25B6%25E7%25BB%25BF%25E4%25BD%2593%25E5%259F%25BA%25E5%259B%25A0%25E7%25BB%2584%25E6%2595%25B0%25E6%258D%25AE\\%25E7%25BB%2584%25E8%25A3%2585%25E7%259A%2584%25E5%258F%25B6%25E7%25BB%25BF%25E4%25BD%2593%25E4%25BF%25A1%25E6%2581%25AF\\CL\\CPGAVAS2\\166073366105132.fas.2.7.7.80.10.50.500.1.txt.html#82578--82623,24,1.9,24,12) | 24 | 1.9 | 24 | 86 | 1.95 |
|  | [84,886—84,923](file:///D:\\%25E6%25A1%258C%25E9%259D%25A2\\%25E5%258F%25B6%25E7%25BB%25BF%25E4%25BD%2593%25E5%259F%25BA%25E5%259B%25A0%25E7%25BB%2584%25E6%2595%25B0%25E6%258D%25AE\\%25E7%25BB%2584%25E8%25A3%2585%25E7%259A%2584%25E5%258F%25B6%25E7%25BB%25BF%25E4%25BD%2593%25E4%25BF%25A1%25E6%2581%25AF\\CL\\CPGAVAS2\\166073366105132.fas.2.7.7.80.10.50.500.1.txt.html#84886--84923,17,2.2,17,13) | 17 | 2.2 | 17 | 90 | 1.73 |
|  | [92,228—92,279](file:///D:\\%25E6%25A1%258C%25E9%259D%25A2\\%25E5%258F%25B6%25E7%25BB%25BF%25E4%25BD%2593%25E5%259F%25BA%25E5%259B%25A0%25E7%25BB%2584%25E6%2595%25B0%25E6%258D%25AE\\%25E7%25BB%2584%25E8%25A3%2585%25E7%259A%2584%25E5%258F%25B6%25E7%25BB%25BF%25E4%25BD%2593%25E4%25BF%25A1%25E6%2581%25AF\\CL\\CPGAVAS2\\166073366105132.fas.2.7.7.80.10.50.500.1.txt.html#92228--92279,21,2.5,21,14) | 21 | 2.5 | 21 | 100 | 1.63 |
|  | [92,208—92,296](file:///D:\\%25E6%25A1%258C%25E9%259D%25A2\\%25E5%258F%25B6%25E7%25BB%25BF%25E4%25BD%2593%25E5%259F%25BA%25E5%259B%25A0%25E7%25BB%2584%25E6%2595%25B0%25E6%258D%25AE\\%25E7%25BB%2584%25E8%25A3%2585%25E7%259A%2584%25E5%258F%25B6%25E7%25BB%25BF%25E4%25BD%2593%25E4%25BF%25A1%25E6%2581%25AF\\CL\\CPGAVAS2\\166073366105132.fas.2.7.7.80.10.50.500.1.txt.html#92208--92296,21,4.1,21,15) | 21 | 4.1 | 21 | 84 | 1.68 |
|  | [94,254—94,283](file:///D:\\%25E6%25A1%258C%25E9%259D%25A2\\%25E5%258F%25B6%25E7%25BB%25BF%25E4%25BD%2593%25E5%259F%25BA%25E5%259B%25A0%25E7%25BB%2584%25E6%2595%25B0%25E6%258D%25AE\\%25E7%25BB%2584%25E8%25A3%2585%25E7%259A%2584%25E5%258F%25B6%25E7%25BB%25BF%25E4%25BD%2593%25E4%25BF%25A1%25E6%2581%25AF\\CL\\CPGAVAS2\\166073366105132.fas.2.7.7.80.10.50.500.1.txt.html#94254--94283,15,2.0,15,16) | 15 | 2.0 | 15 | 100 | 1.80 |
|  | [94,662—94,733](file:///D:\\%25E6%25A1%258C%25E9%259D%25A2\\%25E5%258F%25B6%25E7%25BB%25BF%25E4%25BD%2593%25E5%259F%25BA%25E5%259B%25A0%25E7%25BB%2584%25E6%2595%25B0%25E6%258D%25AE\\%25E7%25BB%2584%25E8%25A3%2585%25E7%259A%2584%25E5%258F%25B6%25E7%25BB%25BF%25E4%25BD%2593%25E4%25BF%25A1%25E6%2581%25AF\\CL\\CPGAVAS2\\166073366105132.fas.2.7.7.80.10.50.500.1.txt.html#94662--94733,18,4.2,18,18) | 18 | 4.2 | 18 | 91 | 1.82 |
|  | [102,541—102,575](file:///D:\\%25E6%25A1%258C%25E9%259D%25A2\\%25E5%258F%25B6%25E7%25BB%25BF%25E4%25BD%2593%25E5%259F%25BA%25E5%259B%25A0%25E7%25BB%2584%25E6%2595%25B0%25E6%258D%25AE\\%25E7%25BB%2584%25E8%25A3%2585%25E7%259A%2584%25E5%258F%25B6%25E7%25BB%25BF%25E4%25BD%2593%25E4%25BF%25A1%25E6%2581%25AF\\CL\\CPGAVAS2\\166073366105132.fas.2.7.7.80.10.50.500.1.txt.html#102541--102575,14,2.4,14,19) | 14 | 2.4 | 14 | 90 | 1.63 |
|  | [107,001—107,075](file:///D:\\%25E6%25A1%258C%25E9%259D%25A2\\%25E5%258F%25B6%25E7%25BB%25BF%25E4%25BD%2593%25E5%259F%25BA%25E5%259B%25A0%25E7%25BB%2584%25E6%2595%25B0%25E6%258D%25AE\\%25E7%25BB%2584%25E8%25A3%2585%25E7%259A%2584%25E5%258F%25B6%25E7%25BB%25BF%25E4%25BD%2593%25E4%25BF%25A1%25E6%2581%25AF\\CL\\CPGAVAS2\\166073366105132.fas.2.7.7.80.10.50.500.1.txt.html#107001--107075,38,2.0,37,20) | 38 | 2.0 | 37 | 97 | 1.97 |
|  | [110,591—110,656](file:///D:\\%25E6%25A1%258C%25E9%259D%25A2\\%25E5%258F%25B6%25E7%25BB%25BF%25E4%25BD%2593%25E5%259F%25BA%25E5%259B%25A0%25E7%25BB%2584%25E6%2595%25B0%25E6%258D%25AE\\%25E7%25BB%2584%25E8%25A3%2585%25E7%259A%2584%25E5%258F%25B6%25E7%25BB%25BF%25E4%25BD%2593%25E4%25BF%25A1%25E6%2581%25AF\\CL\\CPGAVAS2\\166073366105132.fas.2.7.7.80.10.50.500.1.txt.html#110591--110656,32,2.1,32,21) | 32 | 2.1 | 32 | 97 | 1.84 |
|  | [116,640—116,682](file:///D:\\%25E6%25A1%258C%25E9%259D%25A2\\%25E5%258F%25B6%25E7%25BB%25BF%25E4%25BD%2593%25E5%259F%25BA%25E5%259B%25A0%25E7%25BB%2584%25E6%2595%25B0%25E6%258D%25AE\\%25E7%25BB%2584%25E8%25A3%2585%25E7%259A%2584%25E5%258F%25B6%25E7%25BB%25BF%25E4%25BD%2593%25E4%25BF%25A1%25E6%2581%25AF\\CL\\CPGAVAS2\\166073366105132.fas.2.7.7.80.10.50.500.1.txt.html#116640--116682,21,2.0,21,23) | 21 | 2.0 | 21 | 100 | 1.57 |
|  | [121,506—121,535](file:///D:\\%25E6%25A1%258C%25E9%259D%25A2\\%25E5%258F%25B6%25E7%25BB%25BF%25E4%25BD%2593%25E5%259F%25BA%25E5%259B%25A0%25E7%25BB%2584%25E6%2595%25B0%25E6%258D%25AE\\%25E7%25BB%2584%25E8%25A3%2585%25E7%259A%2584%25E5%258F%25B6%25E7%25BB%25BF%25E4%25BD%2593%25E4%25BF%25A1%25E6%2581%25AF\\CL\\CPGAVAS2\\166073366105132.fas.2.7.7.80.10.50.500.1.txt.html#121506--121535,15,2.0,15,26) | 15 | 2.0 | 15 | 93 | 1.88 |
|  | [127,516—127,549](file:///D:\\%25E6%25A1%258C%25E9%259D%25A2\\%25E5%258F%25B6%25E7%25BB%25BF%25E4%25BD%2593%25E5%259F%25BA%25E5%259B%25A0%25E7%25BB%2584%25E6%2595%25B0%25E6%258D%25AE\\%25E7%25BB%2584%25E8%25A3%2585%25E7%259A%2584%25E5%258F%25B6%25E7%25BB%25BF%25E4%25BD%2593%25E4%25BF%25A1%25E6%2581%25AF\\CL\\CPGAVAS2\\166073366105132.fas.2.7.7.80.10.50.500.1.txt.html#127516--127549,15,2.2,16,27) | 15 | 2.2 | 16 | 89 | 1.33 |
|  | [134,373—134,438](file:///D:\\%25E6%25A1%258C%25E9%259D%25A2\\%25E5%258F%25B6%25E7%25BB%25BF%25E4%25BD%2593%25E5%259F%25BA%25E5%259B%25A0%25E7%25BB%2584%25E6%2595%25B0%25E6%258D%25AE\\%25E7%25BB%2584%25E8%25A3%2585%25E7%259A%2584%25E5%258F%25B6%25E7%25BB%25BF%25E4%25BD%2593%25E4%25BF%25A1%25E6%2581%25AF\\CL\\CPGAVAS2\\166073366105132.fas.2.7.7.80.10.50.500.1.txt.html#134373--134438,32,2.1,32,28) | 32 | 2.1 | 32 | 97 | 1.84 |
|  | [137,954—138,028](file:///D:\\%25E6%25A1%258C%25E9%259D%25A2\\%25E5%258F%25B6%25E7%25BB%25BF%25E4%25BD%2593%25E5%259F%25BA%25E5%259B%25A0%25E7%25BB%2584%25E6%2595%25B0%25E6%258D%25AE\\%25E7%25BB%2584%25E8%25A3%2585%25E7%259A%2584%25E5%258F%25B6%25E7%25BB%25BF%25E4%25BD%2593%25E4%25BF%25A1%25E6%2581%25AF\\CL\\CPGAVAS2\\166073366105132.fas.2.7.7.80.10.50.500.1.txt.html#137954--138028,38,2.0,37,29) | 38 | 2.0 | 37 | 97 | 1.97 |
|  | [142,454—142,488](file:///D:\\%25E6%25A1%258C%25E9%259D%25A2\\%25E5%258F%25B6%25E7%25BB%25BF%25E4%25BD%2593%25E5%259F%25BA%25E5%259B%25A0%25E7%25BB%2584%25E6%2595%25B0%25E6%258D%25AE\\%25E7%25BB%2584%25E8%25A3%2585%25E7%259A%2584%25E5%258F%25B6%25E7%25BB%25BF%25E4%25BD%2593%25E4%25BF%25A1%25E6%2581%25AF\\CL\\CPGAVAS2\\166073366105132.fas.2.7.7.80.10.50.500.1.txt.html#142454--142488,14,2.4,14,30) | 14 | 2.4 | 14 | 90 | 1.63 |
|  | [150,296—150,355](file:///D:\\%25E6%25A1%258C%25E9%259D%25A2\\%25E5%258F%25B6%25E7%25BB%25BF%25E4%25BD%2593%25E5%259F%25BA%25E5%259B%25A0%25E7%25BB%2584%25E6%2595%25B0%25E6%258D%25AE\\%25E7%25BB%2584%25E8%25A3%2585%25E7%259A%2584%25E5%258F%25B6%25E7%25BB%25BF%25E4%25BD%2593%25E4%25BF%25A1%25E6%2581%25AF\\CL\\CPGAVAS2\\166073366105132.fas.2.7.7.80.10.50.500.1.txt.html#150296--150355,18,3.3,18,31) | 18 | 3.3 | 18 | 95 | 1.86 |
|  | [150,296—150,354](file:///D:\\%25E6%25A1%258C%25E9%259D%25A2\\%25E5%258F%25B6%25E7%25BB%25BF%25E4%25BD%2593%25E5%259F%25BA%25E5%259B%25A0%25E7%25BB%2584%25E6%2595%25B0%25E6%258D%25AE\\%25E7%25BB%2584%25E8%25A3%2585%25E7%259A%2584%25E5%258F%25B6%25E7%25BB%25BF%25E4%25BD%2593%25E4%25BF%25A1%25E6%2581%25AF\\CL\\CPGAVAS2\\166073366105132.fas.2.7.7.80.10.50.500.1.txt.html#150296--150354,9,6.6,9,32) | 9 | 6.6 | 9 | 72 | 1.83 |
|  | [150,303—150,367](file:///D:\\%25E6%25A1%258C%25E9%259D%25A2\\%25E5%258F%25B6%25E7%25BB%25BF%25E4%25BD%2593%25E5%259F%25BA%25E5%259B%25A0%25E7%25BB%2584%25E6%2595%25B0%25E6%258D%25AE\\%25E7%25BB%2584%25E8%25A3%2585%25E7%259A%2584%25E5%258F%25B6%25E7%25BB%25BF%25E4%25BD%2593%25E4%25BF%25A1%25E6%2581%25AF\\CL\\CPGAVAS2\\166073366105132.fas.2.7.7.80.10.50.500.1.txt.html#150303--150367,18,3.8,17,33) | 18 | 3.8 | 17 | 92 | 1.84 |
|  | [150,746—150,775](file:///D:\\%25E6%25A1%258C%25E9%259D%25A2\\%25E5%258F%25B6%25E7%25BB%25BF%25E4%25BD%2593%25E5%259F%25BA%25E5%259B%25A0%25E7%25BB%2584%25E6%2595%25B0%25E6%258D%25AE\\%25E7%25BB%2584%25E8%25A3%2585%25E7%259A%2584%25E5%258F%25B6%25E7%25BB%25BF%25E4%25BD%2593%25E4%25BF%25A1%25E6%2581%25AF\\CL\\CPGAVAS2\\166073366105132.fas.2.7.7.80.10.50.500.1.txt.html#150746--150775,15,2.0,15,34) | 15 | 2.0 | 15 | 100 | 1.80 |
|  | [152,750—152,801](file:///D:\\%25E6%25A1%258C%25E9%259D%25A2\\%25E5%258F%25B6%25E7%25BB%25BF%25E4%25BD%2593%25E5%259F%25BA%25E5%259B%25A0%25E7%25BB%2584%25E6%2595%25B0%25E6%258D%25AE\\%25E7%25BB%2584%25E8%25A3%2585%25E7%259A%2584%25E5%258F%25B6%25E7%25BB%25BF%25E4%25BD%2593%25E4%25BF%25A1%25E6%2581%25AF\\CL\\CPGAVAS2\\166073366105132.fas.2.7.7.80.10.50.500.1.txt.html#152750--152801,21,2.5,21,36) | 21 | 2.5 | 21 | 100 | 1.63 |

**Table S3.** Forward and Palindromic repeats in the four *Ribes* taxa

| **Species** | **Sequence length** | **First repeat start position** | **Second repeat start position** | **Repetitive type** | **Repetitive Distance** |
| --- | --- | --- | --- | --- | --- |
| Red/White currant  (*Ribes rubrum*) | 46 | 10,742 | 10,742 | P | 0 |
|  | 46 | 94,681 | 150,426 | P | -3 |
|  | 46 | 94,699 | 150,444 | P | -3 |
|  | 42 | 123,815 | 143,461 | P | -1 |
|  | 41 | 30,224 | 30,224 | P | -1 |
|  | 36 | 46,257 | 143,462 | P | -3 |
|  | 34 | 110,597 | 134,508 | P | -1 |
|  | 34 | 110,629 | 134,540 | P | -1 |
|  | 34 | 94,693 | 150,426 | P | -2 |
|  | 34 | 94,711 | 150,444 | P | -2 |
|  | 31 | 107,017 | 138,085 | P | -2 |
|  | 31 | 107,055 | 138,123 | P | -2 |
|  | 30 | 8,940 | 47,950 | P | 0 |
|  | 30 | 17,517 | 17,517 | P | -2 |
|  | 30 | 37,812 | 47,950 | P | -3 |
|  | 30 | 78,891 | 123,818 | P | -3 |
|  | 46 | 94,681 | 94,699 | F | -3 |
|  | 46 | 150,426 | 150,444 | F | -3 |
|  | 42 | 101,668 | 123,815 | F | -1 |
|  | 39 | 46,254 | 123,817 | F | -3 |
|  | 36 | 46,257 | 101,673 | F | -3 |
|  | 34 | 110,597 | 110,629 | F | -1 |
|  | 34 | 134,508 | 134,540 | F | -1 |
|  | 34 | 94,693 | 94,711 | F | -2 |
|  | 32 | 8,938 | 37,810 | F | -3 |
|  | 31 | 107,017 | 107,055 | F | -2 |
|  | 31 | 138,085 | 138,123 | F | -2 |
|  | 30 | 41,306 | 43,530 | F | -3 |
| Blackcurrant  (*Ribes nigrum*) | 46 | 10,487 | 10,487 | P | 0 |
|  | 46 | 94,391 | 150,081 | P | -3 |
|  | 46 | 94,409 | 150,099 | P | -3 |
|  | 42 | 123,579 | 143,123 | P | -1 |
|  | 41 | 29,965 | 29,965 | P | -1 |
|  | 36 | 45,872 | 143,124 | P | -3 |
|  | 34 | 110,302 | 134,168 | P | -1 |
|  | 34 | 110,334 | 134,200 | P | -1 |
|  | 34 | 94,403 | 150,081 | P | -2 |
|  | 34 | 94,421 | 150,099 | P | -2 |
|  | 31 | 91,945 | 152,539 | P | 0 |
|  | 31 | 91,966 | 152,560 | P | 0 |
|  | 31 | 106,722 | 137,745 | P | -2 |
|  | 31 | 106,760 | 137,783 | P | -2 |
|  | 30 | 8,696 | 47,565 | P | 0 |
|  | 30 | 37,463 | 47,565 | P | -3 |
|  | 30 | 78,584 | 123,582 | P | -3 |
|  | 46 | 94,391 | 94,409 | F | -3 |
|  | 46 | 150,081 | 150,099 | F | -3 |
|  | 42 | 101,371 | 123,579 | F | -1 |
|  | 39 | 45,869 | 123,581 | F | -3 |
|  | 36 | 45,872 | 101,376 | F | -3 |
|  | 34 | 110,302 | 110,334 | F | -1 |
|  | 34 | 134,168 | 134,200 | F | -1 |
|  | 34 | 94,403 | 94,421 | F | -2 |
|  | 32 | 8,694 | 37,461 | F | -3 |
|  | 31 | 91,945 | 91,966 | F | 0 |
|  | 31 | 152,539 | 152,560 | F | 0 |
|  | 31 | 106,722 | 106,760 | F | -2 |
|  | 31 | 137,745 | 137,783 | F | -2 |
|  | 30 | 40,941 | 43,165 | F | -3 |
| Gooseberry  (*Ribes uva-crispa*) | 46 | 10,886 | 10,886 | P | 0 |
|  | 46 | 94,673 | 150,291 | P | -3 |
|  | 46 | 94,691 | 150,309 | P | -3 |
|  | 42 | 123,688 | 143,327 | P | -1 |
|  | 41 | 30,356 | 30,356 | P | -1 |
|  | 36 | 46,242 | 143,328 | P | -3 |
|  | 34 | 110,590 | 134,372 | P | -1 |
|  | 34 | 110,622 | 134,404 | P | -1 |
|  | 34 | 94,685 | 150,291 | P | -2 |
|  | 34 | 94,703 | 150,309 | P | -2 |
|  | 31 | 92,227 | 152,749 | P | 0 |
|  | 31 | 92,248 | 152,770 | P | 0 |
|  | 31 | 107,010 | 137,949 | P | -2 |
|  | 31 | 107,048 | 137,987 | P | -2 |
|  | 30 | 9,109 | 47,925 | P | 0 |
|  | 30 | 37,837 | 47,925 | P | -3 |
|  | 30 | 78,882 | 123,691 | P | -3 |
|  | 46 | 94,673 | 94,691 | F | -3 |
|  | 46 | 150,291 | 150,309 | F | -3 |
|  | 42 | 101,659 | 123,688 | F | -1 |
|  | 39 | 46,239 | 123,690 | F | -3 |
|  | 36 | 46,242 | 101,664 | F | -3 |
|  | 34 | 110,590 | 110,622 | F | -1 |
|  | 34 | 134,372 | 134,404 | F | -1 |
|  | 34 | 94,685 | 94,703 | F | -2 |
|  | 32 | 9,107 | 37,835 | F | -3 |
|  | 31 | 92,227 | 92,248 | F | 0 |
|  | 31 | 152,749 | 152,770 | F | 0 |
|  | 31 | 107,010 | 107,048 | F | -2 |
|  | 31 | 137,949 | 137,987 | F | -2 |
|  | 31 | 5,058 | 5,078 | F | -3 |
|  | 30 | 5,027 | 5,056 | F | -1 |
|  | 30 | 5,033 | 5,082 | F | -3 |
|  | 30 | 41,304 | 43,528 | F | -3 |

**Table S4.** SSR loci identified in the four *Ribes* chloroplast genomes

| **Species** | **Repetitive sequence** | **Length** | **Initiation site** | **Termination site** | **Position** | **Location** |
| --- | --- | --- | --- | --- | --- | --- |
| Blackcurrant  (*Ribes nigrum*) | (A)10 | 10 | 20 | 29 | *trnH-GUG* | LSC |
|  | (A)10 | 10 | 3,987 | 3,996 | *trnK-UUU*-intron 1 | LSC |
|  | (A)10 | 10 | 4,504 | 4,513 | IGS (*trnK-UUU*, *rps16*) | LSC |
|  | (T)12 | 12 | 4,617 | 4,628 | IGS (*trnK-UUU*, *rps16*) | LSC |
|  | (A)10 | 10 | 5,626 | 5,635 | *rps16*-intron 1 | LSC |
|  | (T)11 | 11 | 6,661 | 6,671 | IGS (*rps16*, *trnQ-UUG*) | LSC |
|  | (A)13 | 13 | 9,047 | 9,059 | IGS (*trnS-GCU*, *trnG-GCC*) | LSC |
|  | (T)12 | 12 | 9,439 | 9,450 | IGS (*trnS-GCU*, *trnG-GCC*) | LSC |
|  | (T)13 | 13 | 10,380 | 10,392 | *trnG-GCC*-intron 1 | LSC |
|  | (A)15 | 15 | 12,597 | 12,611 | *atpF*-exon 2 | LSC |
|  | (T)13 | 13 | 14,026 | 14,038 | IGS (*atpF*, *atpH*) | LSC |
|  | (T)13 | 13 | 17,273 | 17,285 | IGS (*rps2*, *rpoC2*) | LSC |
|  | (A)10 | 10 | 17,287 | 17,296 | IGS (*rps2*, *rpoC2*) | LSC |
|  | (A)12 | 12 | 17,566 | 17,577 | *rpoC2* | LSC |
|  | (T)10 | 10 | 27,276 | 27,285 | *rpoB* | LSC |
|  | (T)13 | 13 | 29,415 | 29,427 | IGS (*trnC-GCA*, *petN*) | LSC |
|  | (T)11 | 11 | 31,312 | 31,322 | IGS (*psbM*, *trnD-GUC*) | LSC |
|  | (A)12 | 12 | 32,344 | 32,355 | *trnD-GUC* | LSC |
|  | (A)10 | 10 | 34,531 | 34,540 | IGS (*trnT-GGU*, *psbD*) | LSC |
|  | (T)12 | 12 | 37,444 | 37,455 | IGS (*psbC*, *trnS-UGA*) | LSC |
|  | (A)10 | 10 | 37,827 | 37,836 | IGS (*trnS-UGA*, *psbZ*) | LSC |
|  | (A)12 | 12 | 53,614 | 53,625 | IGS (*ndhC*, *trnV-UAC*) | LSC |
|  | (T)11 | 11 | 53,780 | 53,790 | IGS (*ndhC*, *trnV-UAC*) | LSC |
|  | (T)10 | 10 | 54,376 | 54,385 | *trnV-UAC*-intron 1 | LSC |
|  | (T)12 | 12 | 54,656 | 54,667 | *trnV-UAC*-intron 1 | LSC |
|  | (A)14 | 14 | 57,734 | 57,747 | IGS (*atpB*, *rbcL*) | LSC |
|  | (T)11 | 11 | 61,719 | 61,729 | IGS (*accD*, *psaI*) | LSC |
|  | (A)14 | 14 | 63,918 | 63,931 | *cemA* | LSC |
|  | (T)10 | 10 | 66,302 | 66,311 | IGS (*petA*, *psbJ*) | LSC |
|  | (T)10 | 10 | 68,085 | 68,094 | IGS (*psbE*, *petL*) | LSC |
|  | (T)10 | 10 | 69,158 | 69,167 | *petG* | LSC |
|  | (T)11 | 11 | 69,315 | 69,325 | IGS (*petG*, *trnW-CCA*) | LSC |
|  | (T)12 | 12 | 72,165 | 72,176 | IGS (*rpl20*, *rps12/rps12-2*) | LSC |
|  | (A)10 | 10 | 73,787 | 73,796 | *clpP*-intron 2 | LSC |
|  | (A)11 | 11 | 74,274 | 74,284 | *clpP*-intron 1 | LSC |
|  | (T)10 | 10 | 77,062 | 77,071 | IGS (*psbB*, *psbT*) | LSC |
|  | (A)12 | 12 | 80,928 | 80,939 | *rpoA* | LSC |
|  | (T)10 | 10 | 83,107 | 83,116 | *rps8* | LSC |
|  | (A)11 | 11 | 84,146 | 84,156 | *rpl16*-exon 2 | LSC |
|  | (T)10 | 10 | 85,554 | 85,563 | IGS (*rpl16*, *rps3*) | LSC |
|  | (T)10 | 10 | 87,266 | 87,275 | *rpl2*-exon 2 | IRb |
|  | (T)12 | 12 | 105,835 | 105,846 | *trnI-GAU*-intron 1 | IRb |
|  | (A)13 | 13 | 110,783 | 110,795 | IGS (*rrn5S*, *trnR-ACG*) | IRb |
|  | (T)10 | 10 | 111,254 | 111,263 | IGS (*trnR-ACG*, *trnN-GUU*) | IRb |
|  | (T)11 | 11 | 113,610 | 113,620 | *ndhF* | SSC |
|  | (A)10 | 10 | 115,825 | 115,834 | IGS (*ndhF*, *rpl32*) | SSC |
|  | (A)10 | 10 | 116,081 | 116,090 | IGS (*ndhF*, *rpl32*) | SSC |
|  | (T)14 | 14 | 117,751 | 117,764 | *ccsA* | SSC |
|  | (A)12 | 12 | 125,846 | 125,857 | *ndhH* | SSC |
|  | (T)10 | 10 | 127,438 | 127,447 | *ycf1* | SSC |
|  | (T)11 | 11 | 127,625 | 127,635 | *ycf1* | SSC |
|  | (A)10 | 10 | 133,482 | 133,491 | IGS (*trnN-GUU*-2, *trnR-ACG-2*) | IRa |
|  | (T)13 | 13 | 133,950 | 133,962 | *rrn5S-2* | IRa |
|  | (A)12 | 12 | 138,899 | 138,910 | *trnI-GAU-2*-intron 1 | IRa |
| Red/White currant  (*Ribes rubrum*) | (A)10 | 10 | 3,875 | 3,884 | *trnK-UUU*-intron 1 | LSC |
|  | (A)10 | 10 | 4,398 | 4,407 | IGS (*trnK-UUU*, *rps16*) | LSC |
|  | (T)11 | 11 | 4,511 | 4,521 | IGS (*trnK-UUU*, *rps16*) | LSC |
|  | (A)11 | 11 | 4,701 | 4,711 | IGS (*trnK-UUU*, *rps16*) | LSC |
|  | (T)11 | 11 | 9,586 | 9,596 | IGS (*trnS-GCU*, *trnG-GCC*) | LSC |
|  | (T)10 | 10 | 10,526 | 10,535 | *trnG-GCC*-intron 1 | LSC |
|  | (A)10 | 10 | 11,115 | 11,124 | IGS (*trnR-UCU*, *atpA*) | LSC |
|  | (A)14 | 14 | 12,757 | 12,770 | IGS (*atpA*, *atpF*) | LSC |
|  | (T)11 | 11 | 14,185 | 14,195 | IGS (*atpF*, *atpH*) | LSC |
|  | (T)11 | 11 | 17,522 | 17,533 | IGS (*rps2*, *rpoC2*) | LSC |
|  | (A)10 | 10 | 17,535 | 17,545 | IGS (*rps2*, *rpoC2*) | LSC |
|  | (T)10 | 10 | 27,426 | 27,435 | *rpoB* | LSC |
|  | (T)10 | 10 | 29,573 | 29,582 | IGS (*trnC-GCA*, *petN*) | LSC |
|  | (T)10 | 10 | 31,469 | 31,478 | IGS (*psbM*, *trnD-GUC*) | LSC |
|  | (A)10 | 10 | 32,505 | 32,514 | IGS (*psbM*, *trnD-GUC*) | LSC |
|  | (AT)6 | 12 | 34,460 | 34,471 | IGS (*trnT-GGU*, *psbD*) | LSC |
|  | (A)12 | 12 | 34,754 | 34,765 | IGS (*trnT-GGU*, *psbD*) | LSC |
|  | (TA)6 | 12 | 44,886 | 44,897 | IGS (*psaA*, *ycf3*) | LSC |
|  | (T)10 | 10 | 48,195 | 48,204 | IGS (*trnS-GGA*, *r*ps4) | LSC |
|  | (T)11 | 11 | 54,042 | 54,052 | IGS (*ndhC*, *trnV-UAC*) | LSC |
|  | (T)10 | 10 | 54,910 | 54,919 | *trnV-UAC*-intron 1 | LSC |
|  | (A)13 | 13 | 57,984 | 57,996 | IGS (*atpB*, *rbcL*) | LSC |
|  | (T)11 | 11 | 61,934 | 61,944 | IGS (*accD*, *psaI*) | LSC |
|  | (A)11 | 11 | 64,144 | 64,154 | *cemA* | LSC |
|  | (T)14 | 14 | 66,540 | 66,553 | IGS (*petA*, *psbJ*) | LSC |
|  | (T)10 | 10 | 68,325 | 68,334 | IGS (*psbE*, *petL*) | LSC |
|  | (T)10 | 10 | 69,394 | 69,403 | IGS (*petL*, *petG*) | LSC |
|  | (T)10 | 10 | 72,392 | 72,401 | IGS (*rpl20*, *rps12/rps12-2*) | LSC |
|  | (A)11 | 11 | 74,012 | 74,022 | *clpP*-intron 2 | LSC |
|  | (T)10 | 10 | 77,285 | 77,294 | IGS (*psbB*, *psbT*) | LSC |
|  | (T)10 | 10 | 83,301 | 83,310 | IGS (*infA*, *rps8*) | LSC |
|  | (T)11 | 11 | 85,742 | 85,752 | *rpl16*-intron 1 | LSC |
|  | (T)10 | 10 | 106,028 | 106,037 | *trnI-GAU*-intron 1 | IRb |
|  | (T)11 | 11 | 111,440 | 111,450 | IGS (*trnR-ACG*, *trnN-GUU*) | IRb |
|  | (A)10 | 10 | 117,272 | 117,281 | IGS (*rpl32*, *trnL-UAG*) | SSC |
|  | (A)13 | 13 | 127,115 | 127,127 | IGS (*rps15*, *ycf1*) | SSC |
|  | (T)10 | 10 | 128,901 | 128,910 | *ycf1* | SSC |
|  | (A)11 | 11 | 131,365 | 131,375 | *ycf1* | SSC |
|  | (A)11 | 11 | 133,722 | 133,732 | IGS (*trnN-GUU*-2, *trnR-ACG*-2) | IRa |
|  | (A)10 | 10 | 139,135 | 139,144 | *trnI-GAU-2*-intron 1 | IRa |
| Gooseberry  (*Ribes uva-crispa*) | (T)10 | 10 | 4,525 | 4,534 | IGS (*trnK-UUU*, *rps16*) | LSC |
|  | (T)10 | 10 | 6,415 | 6,424 | IGS (*rps16*, *trnQ-UUG*) | LSC |
|  | (A)11 | 11 | 6,703 | 6,713 | IGS (*rps16*, *trnQ-UUG*) | LSC |
|  | (T)11 | 11 | 9,742 | 9,752 | IGS (*trnS-GCU*, *trnG-GCC*) | LSC |
|  | (T)10 | 10 | 10,682 | 10,691 | *trnG-GCC*-intron 1 | LSC |
|  | (A)10 | 10 | 11,251 | 11,260 | IGS (*trnR-UCU*, *atpA*) | LSC |
|  | (A)20 | 20 | 12,891 | 12,910 | IGS (*atpA*, *atpF*) | LSC |
|  | (T)10 | 10 | 14,325 | 14,334 | IGS (*atpF*, *atpH*) | LSC |
|  | (T)10 | 10 | 15,862 | 15,871 | IGS (*atpH*, *atpI*) | LSC |
|  | (T)11 | 11 | 17,673 | 17,683 | IGS (*rps2*, *rpoC2*) | LSC |
|  | (A)11 | 11 | 17,857 | 17,867 | IGS (*rps2*, *rpoC2*) | LSC |
|  | (T)10 | 10 | 27,566 | 27,575 | *rpoB* | LSC |
|  | (T)10 | 10 | 29,201 | 29,210 | IGS (*rpoB*, *trnC-GCA*) | LSC |
|  | (T)13 | 13 | 29,702 | 29,714 | IGS (*trnC-GCA*, *petN*) | LSC |
|  | (T)10 | 10 | 37,716 | 37,725 | IGS (*psbC*, *trnS-UGA*) | LSC |
|  | (A)12 | 12 | 38,097 | 38,108 | IGS (*trnS-UGA*, *psbZ*) | LSC |
|  | (A)11 | 11 | 39,929 | 39,939 | IGS (*rps14*, *psaB*) | LSC |
|  | (TA)6 | 12 | 44,873 | 44,885 | IGS (*psaA*, *ycf3*) | LSC |
|  | (T)11 | 10 | 44,886 | 44,897 | IGS (*psaA*, *ycf3*) | LSC |
|  | (A)10 | 10 | 47,150 | 47,159 | IGS (*ycf3*, *trnS-GGA*) | LSC |
|  | (T)10 | 10 | 51,449 | 51,458 | IGS (*trnF-GAA*, *ndhJ*) | LSC |
|  | (T)11 | 11 | 54,023 | 54,033 | IGS (*ndhC*, *trnV-UAC*) | LSC |
|  | (T)10 | 10 | 54,896 | 54,905 | *trnV-UAC*-intron 1 | LSC |
|  | (A)10 | 10 | 57,970 | 57,979 | IGS (*atpB*, *rbcL*) | LSC |
|  | (T)10 | 10 | 61,943 | 61,952 | IGS (*accD*, *psaI*) | LSC |
|  | (T)11 | 11 | 68,306 | 68,316 | IGS (*psbE*, *petL*) | LSC |
|  | (T)11 | 11 | 69,369 | 69,379 | IGS (*petL*, *petG*) | LSC |
|  | (A)11 | 11 | 73,996 | 74,006 | *clpP*-intron 2 | LSC |
|  | (T)11 | 11 | 77,270 | 77,280 | IGS (*psbB*, *psbT*) | LSC |
|  | (T)10 | 10 | 83,298 | 83,307 | IGS (*infA*, *rps8*) | LSC |
|  | (A)11 | 11 | 84,332 | 84,342 | IGS (*rpl14*, *rpl16*) | LSC |
|  | (T)10 | 10 | 85,733 | 85,742 | *rpl16*-intron 1 | LSC |
|  | (T)12 | 12 | 106,019 | 106,030 | *trnI-GAU*-intron 1 | IRb |
|  | (A)11 | 11 | 110,967 | 110,977 | IGS (*rrn5S*, *trnR-ACG*) | IRb |
|  | (T)11 | 11 | 111,436 | 111,446 | IGS (*trnR-ACG*, *trnN-GUU*) | IRb |
|  | (A)10 | 10 | 117,303 | 117,312 | IGS (*rpl32*, *trnL-UAG*) | SSC |
|  | (T)11 | 11 | 118,894 | 118,904 | IGS (*ccsA*, *ndhD*) | SSC |
|  | (T)10 | 10 | 128,759 | 128,768 | *ycf1* | SSC |
|  | (A)11 | 11 | 131,226 | 131,236 | *ycf1* | SSC |
|  | (A)11 | 11 | 133,583 | 133,593 | IGS (*trnN-GUU*-2, *trnR-ACG*-2) | IRa |
|  | (T)11 | 11 | 134,052 | 134,062 | IGS (*trnR-ACG*-2, *rrn5S*-2) | IRa |
|  | (A)12 | 12 | 138,999 | 139,010 | *trnI-GAU-2*-intron 1 | IRa |

**Table S5.** Genetic similarity matrix of the nine *Ribes* cp genomes

|  | *Ribes rubrum* | *Ribes rubrum* (white) | *Ribes nigrum* | *Ribes uva-crispa* | *Ribes fasciculatum* var. *chinense* | *Ribes odoratum* | *Ribes nevadense* | *Ribes roezlii* | *Ribes glaciale* |
| --- | --- | --- | --- | --- | --- | --- | --- | --- | --- |
| *Ribes rubrum*  (OP888488) | NA |  |  |  |  |  |  |  |  |
| *Ribes rubrum* (white)  (OP888486) | 1.000 | NA |  |  |  |  |  |  |  |
| *Ribes nigrum*  (OP888489) | 0.985 | 0.985 | NA |  |  |  |  |  |  |
| *Ribes uva-crispa*  (OP888487) | 0.985 | 0.985 | 0.985 | NA |  |  |  |  |  |
| *Ribes fasciculatum* var. *chinense*  (MH191388) | 0.976 | 0.976 | 0.974 | 0.974 | NA |  |  |  |  |
| *Ribes odoratum*  (MT081309) | 0.982 | 0.982 | 0.979 | 0.980 | 0.971 | NA |  |  |  |
| *Ribes nevadense*  (MN496075) | 0.987 | 0.987 | 0.989 | 0.987 | 0.975 | 0.981 | NA |  |  |
| *Ribes roezlii*  (MN496076) | 0.985 | 0.985 | 0.986 | 0.987 | 0.974 | 0.980 | 0.988 | NA |  |
| *Ribes glaciale*  (NC062156) | 0.994 | 0.994 | 0.985 | 0.985 | 0.976 | 0.982 | 0.987 | 0.986 | NA |

**Table S6.** SNPs and Indels identified in the nine *Ribes* chloroplast genomes

| **Feature** | **Location** | **Count** |
| --- | --- | --- |
| Intergenic spacer | IGS (*trnT-GGU*, *psbD*) | 86 |
|  | IGS (*ndhF*, *rpl32*) | 76 |
|  | IGS (*atpH*, *atpI*) | 71 |
|  | IGS (*psbM*, *trnD-GUC*) | 67 |
|  | IGS (*rps16*, *trnQ-UUG*) | 63 |
|  | IGS (*trnK-UUU*, *rps16*) | 58 |
|  | IGS (*trnT-UGU*, *trnL-UAA*) | 58 |
|  | IGS (*rpl32*, *trnL-UAG*) | 58 |
|  | IGS (*petN*, *psbM*) | 56 |
|  | IGS (*psbE*, *petL*) | 52 |
|  | IGS (*rpoB*, *trnC-GCA*) | 48 |
|  | IGS (*trnC-GCA*, *petN*) | 48 |
|  | IGS (*trnS-GCU*, *trnG-GCC*) | 47 |
|  | IGS (*psbZ*, *trnG-UCC*) | 45 |
|  | IGS (*accD*, *psaI*) | 40 |
|  | IGS (*ycf3*, *trnS-GGA*) | 39 |
|  | IGS (*trnF-GAA*, *ndhJ*) | 39 |
|  | IGS (*petA*, *psbJ*) | 38 |
|  | IGS (*ndhC*, *trnV-UAC*) | 37 |
|  | IGS (*trnH-GUG*, *psbA*) | 36 |
|  | IGS (*rps15*, *ycf1-2*) | 34 |
|  | IGS (*psaA*, *ycf3*) | 32 |
|  | IGS (*atpB*, *rbcL*) | 31 |
|  | IGS (*psbK*, *psbI*) | 30 |
|  | IGS (*trnE-UUC*, *trnT-GGU*) | 30 |
|  | IGS (*rpl20*, *rps12*) | 28 |
|  | IGS (*ycf4*, *cemA*) | 27 |
|  | IGS (*atpF*, *atpH*) | 23 |
|  | IGS (*rbcL*, *accD*) | 23 |
|  | IGS (*ccsA*, *ndhD*) | 22 |
|  | IGS (*trnL-UAA*, *trnF-GAA*) | 21 |
|  | IGS (*ndhG*, *ndhI*) | 21 |
|  | IGS (*psaC*, *ndhE*) | 19 |
|  | IGS (*rps12*, *trnV-GAC*) | 18 |
|  | IGS (*trnV-GAC-2*, *rps12-2*) | 18 |
|  | IGS (*trnQ-UUG*, *psbK*) | 17 |
|  | IGS (*trnS-UGA*, *psbZ*) | 17 |
|  | IGS (*psbA*, *trnK-UUU*) | 16 |
|  | IGS (*rps2*, *rpoC2*) | 16 |
|  | IGS (*psaI*, *ycf4*) | 16 |
|  | IGS (*trnP-UGG*, *psaJ*) | 16 |
|  | IGS (*trnR-UCU*, *atpA*) | 15 |
|  | IGS (*trnN-GUU-2*, *trnR-ACG-2*) | 15 |
|  | IGS (*psaJ*, *rpl33*) | 14 |
|  | IGS (*rps18*, *rpl20*) | 14 |
|  | IGS (*rps8*, *rpl14*) | 14 |
|  | IGS (*clpP*, *psbB*) | 13 |
|  | IGS (*trnR-ACG*, *trnN-GUU*) | 13 |
|  | IGS (*trnG-GCC*, *trnR-UCU*) | 12 |
|  | IGS (*trnG-UCC*, *trnfM-CAU*) | 12 |
|  | IGS (*rps14*, *psaB*) | 12 |
|  | IGS (*trnL-UAG*, *ccsA*) | 12 |
|  | IGS (*rps4*, *trnT-UGU*) | 11 |
|  | IGS (*trnM-CAU*, *atpE*) | 11 |
|  | IGS (*trnS-GGA*, *rps4*) | 10 |
|  | IGS (*cemA*, *petA*) | 10 |
|  | IGS (*trnL-CAA*, *ndhB*) | 9 |
|  | IGS (*ndhB-2*, *trnL-CAA-2*) | 9 |
|  | IGS (*trnL-CAA-2*, *ycf2-2*) | 9 |
|  | IGS (*atpI*, *rps2*) | 8 |
|  | IGS (*trnD-GUC*, *trnY-GUA*) | 8 |
|  | IGS (*rpl16*, *rps3*) | 8 |
|  | IGS (*ycf2*, *trnL-CAA*) | 8 |
|  | IGS (*psbC*, *trnS-UGA*) | 7 |
|  | IGS (*trnV-UAC*, *trnM-CAU*) | 7 |
|  | IGS (*petL*, *petG*) | 7 |
|  | IGS (*petG*, *trnW-CCA*) | 7 |
|  | IGS (*rpl14*, *rpl16*) | 7 |
|  | IGS (*rps19*, *rpl2*) | 7 |
|  | IGS (*ndhI*, *ndhA*) | 7 |
|  | IGS (*psbI*, *trnS-GCU*) | 6 |
|  | IGS (*trnW-CCA*, *trnP-UGG*) | 6 |
|  | IGS (*psbB*, *psbT*) | 6 |
|  | IGS (*petD*, *rpoA*) | 6 |
|  | IGS (*rps11*, *rpl36*) | 6 |
|  | IGS (*ndhD*, *psaC*) | 6 |
|  | IGS (*ndhE*, *ndhG*) | 6 |
|  | IGS (*rps12/rps12-2*, *clpP*) | 5 |
|  | IGS (*rrn5*, *trnR-ACG*) | 5 |
|  | IGS (*trnR-ACG-2*, *rrn5-2*) | 5 |
|  | IGS (*rpl2-2*, *trnH-GUG*) | 5 |
|  | IGS (*trnfM-CAU*, *rps14*) | 4 |
|  | IGS (*rpl33*, *rps18*) | 4 |
|  | IGS (*rpl36*, *infA*) | 4 |
|  | IGS (*rpl22*, *rps19*) | 4 |
|  | IGS (*rrn4.5*, *rrn5*) | 4 |
|  | IGS (*rrn5-2*, *rrn4.5-2*) | 4 |
|  | IGS (*atpA*, *atpF*) | 3 |
|  | IGS (*ndhJ*, *ndhK*) | 3 |
|  | IGS (*psbJ*, *psbL*) | 3 |
|  | IGS (*psbT*, *psbN*) | 3 |
|  | IGS (*psbN*, *psbH*) | 3 |
|  | IGS (*psbH*, *petB*) | 3 |
|  | IGS (*petB*, *petD*) | 3 |
|  | IGS (*infA*, *rps8*) | 3 |
|  | IGS (*ndhH*, *rps15*) | 3 |
|  | IGS (*rps7-2*, *ndhB-2*) | 3 |
|  | IGS (*rps3*, *rpl22*) | 2 |
|  | IGS (*ndhB*, *rps7*) | 2 |
|  | IGS (*rrn16*, *trnI-GAU*) | 2 |
|  | IGS (*trnI-GAU-2*, *rrn16-2*) | 2 |
|  | IGS (*rpoC2*, *rpoC1*) | 1 |
|  | IGS (*trnY-GUA*, *trnE-UUC*) | 1 |
|  | IGS (*psbL*, *psbF*) | 1 |
|  | IGS (*rpoA*, *rps11*) | 1 |
|  | IGS (*rps7*, *rps12*) | 1 |
|  | IGS (*trnV-GAC*, *rrn16*) | 1 |
|  | IGS (*trnA-UGC*, *rrn23*) | 1 |
|  | IGS (*rrn23*, *rrn4.5*) | 1 |
|  | IGS (*trnN-GUU*, *ycf1*) | 1 |
|  | IGS (*ycf1-2*, *trnN-GUU-2*) | 1 |
|  | IGS (*rrn4.5-2*, *rrn23-2*) | 1 |
|  | IGS (*rrn23-2*, *trnA-UGC*) | 1 |
|  | IGS (*rrn16-2*, *trnV-GAC-2*) | 1 |
|  | IGS (*rps12-2*, *rps7-2*) | 1 |
| Intron | *trnK-UUU*-intron 1, *matK* | 57 |
|  | *rpl16*-intron 1 | 43 |
|  | *ndhA*-intron 1 | 41 |
|  | *clpP*-intron 1 | 40 |
|  | *trnK-UUU*-intron 1 | 32 |
|  | *atpF*-intron 1 | 32 |
|  | *petB*-intron 1 | 32 |
|  | *rps16*-intron 1 | 30 |
|  | *clpP*-intron 2 | 24 |
|  | *trnG-GCC*-intron 1 | 23 |
|  | *rpoC1*-intron 1 | 23 |
|  | *ycf3*-intron 1 | 21 |
|  | *petD*-intron 1 | 21 |
|  | *ycf3*-intron 2 | 18 |
|  | *trnL-UAA*-intron 1 | 15 |
|  | *trnV-UAC*-intron 1 | 10 |
|  | *trnI-GAU*-intron 1 | 9 |
|  | *trnI-GAU-2*-intron 1 | 8 |
|  | *rpl2*-intron 1 | 5 |
|  | *rpl2-2*-intron 1 | 5 |
|  | *ndhB*-intron 1 | 4 |
|  | *ndhB-2*-intron 1 | 4 |
|  | *trnA-UGC*-intron 1 | 3 |
|  | *rps12*-intron 1 | 2 |
|  | *rps12-2*-intron 1 | 2 |
| CDS | *ycf1-2* | 248 |
|  | *rpoC2* | 104 |
|  | *ycf2* | 56 |
|  | *ndhF* | 56 |
|  | *ycf2-2* | 56 |
|  | *rpoB* | 54 |
|  | *ndhD* | 39 |
|  | *rpoA* | 36 |
|  | *accD* | 34 |
|  | *psaB* | 30 |
|  | *ccsA* | 30 |
|  | *atpB* | 29 |
|  | *psbB* | 27 |
|  | *rbcL* | 26 |
|  | *ndhH* | 26 |
|  | *rpoC1*-exon 2 | 25 |
|  | *psaA* | 23 |
|  | *atpA* | 21 |
|  | *ndhK* | 19 |
|  | *psbC* | 16 |
|  | *atpI* | 14 |
|  | *petA* | 14 |
|  | *ndhI* | 14 |
|  | *ycf4* | 12 |
|  | *ndhG* | 12 |
|  | *psbD* | 11 |
|  | *rps3* | 11 |
|  | *rpl22* | 11 |
|  | *rps19* | 10 |
|  | *ycf1* | 10 |
|  | *ndhA*-exon 2 | 10 |
|  | *ndhA*-exon 1 | 10 |
|  | *psbA* | 9 |
|  | *rpl20* | 9 |
|  | *petB*-exon 2 | 9 |
|  | *atpF*-exon 2 | 8 |
|  | *rps2* | 8 |
|  | *cemA* | 8 |
|  | *petD*-exon 2 | 8 |
|  | *rps11* | 8 |
|  | *ycf1*, *ndhF* | 8 |
|  | *rps4* | 7 |
|  | *atpE* | 7 |
|  | *rpoC1*-exon 1 | 6 |
|  | *rps8* | 6 |
|  | *rpl16*-exon 2 | 6 |
|  | *ndhE* | 6 |
|  | *clpP*-exon 3 | 5 |
|  | *rpl14* | 5 |
|  | *atpH* | 4 |
|  | *ycf3*-exon 3 | 4 |
|  | *rpl33* | 4 |
|  | *rps18* | 4 |
|  | *clpP*-exon 2 | 4 |
|  | *psbT* | 4 |
|  | *rpl2*-exon 2 | 4 |
|  | *ndhB*-exon 2 | 4 |
|  | *rpl32* | 4 |
|  | *rps15* | 4 |
|  | *ndhB-2*-exon 2 | 4 |
|  | *rpl2-2*-exon 2 | 4 |
|  | *psbZ* | 3 |
|  | *ndhJ* | 3 |
|  | *psbJ* | 3 |
|  | *infA* | 3 |
|  | *rpl23* | 3 |
|  | *rpl23-2* | 3 |
|  | *rps16*-exon 2 | 2 |
|  | *psbK* | 2 |
|  | *atpF*-exon 1 | 2 |
|  | *trnC-GCA* | 2 |
|  | *psbM* | 2 |
|  | *rps14* | 2 |
|  | *ycf3*-exon 2 | 2 |
|  | *psbL* | 2 |
|  | *psbF* | 2 |
|  | *petL* | 2 |
|  | *psaJ* | 2 |
|  | *clpP*-exon 1 | 2 |
|  | *psbH* | 2 |
|  | *rps7* | 2 |
|  | *rrn23* | 2 |
|  | *rrn23-2* | 2 |
|  | *psbI* | 1 |
|  | *trnT-UGU* | 1 |
|  | *trnL-UAA*-exon 1 | 1 |
|  | *ndhK*, *ndhC* | 1 |
|  | *ndhC* | 1 |
|  | *psbE* | 1 |
|  | *rps12*-exon 1 | 1 |
|  | *psbN* | 1 |
|  | *rpl36* | 1 |
|  | *ndhB*-exon 1 | 1 |
|  | *rrn16* | 1 |
|  | *trnL-UAG* | 1 |
|  | *psaC* | 1 |
|  | *rrn16-2* | 1 |
|  | *rps7-2* | 1 |
|  | *ndhB-2*-exon 1 | 1 |
|  | *trnL-UAA-*exon 2 | 1 |

**Table S9.** Pairwise comparison of nine *Ribes* chloroplast genome variation sites

|  | *Ribes rubrum* | *Ribes rubrum* (white) | *Ribes nigrum* | *Ribes uva-crispa* | *Ribes fasciculatum* var. *chinense* | *Ribes odoratum* | *Ribes nevadense* | *Ribes roezlii* | *Ribes glaciale* |
| --- | --- | --- | --- | --- | --- | --- | --- | --- | --- |
| *Ribes rubrum*  (OP888488) | NA |  |  |  |  |  |  |  |  |
| *Ribes rubrum* (white)  (OP888486) | 0 | NA |  |  |  |  |  |  |  |
| *Ribes nigrum*  (OP888489) | 983 | 983 | NA |  |  |  |  |  |  |
| *Ribes uva-crispa*  (OP888487) | 1,122 | 1,122 | 851 | NA |  |  |  |  |  |
| *Ribes fasciculatum* var. *chinense*  (MH191388) | 1,608 | 1,608 | 1,642 | 1,489 | NA |  |  |  |  |
| *Ribes odoratum*  (MT081309) | 1,199 | 1,199 | 1,209 | 1,068 | 1,812 | NA |  |  |  |
| *Ribes nevadense*  (MN496075) | 964 | 964 | 507 | 808 | 1,599 | 1,172 | NA |  |  |
| *Ribes roezlii*  (MN496076) | 1,227 | 1,227 | 938 | 941 | 1,863 | 1,417 | 898 | NA |  |
| *Ribes glaciale*  (NC062156) | 573 | 573 | 940 | 1,076 | 1,559 | 1,141 | 901 | 1,176 | NA |

**Table S10.** The 27 species used in phylogenetic analysis and their corresponding GenBank accession number

| **Family** | **Genus** | **Species** | **GenBank No.** |
| --- | --- | --- | --- |
| Grossulariaceae | *Ribes* | *Ribes nevadense* | MN496075 |
|  |  | *Ribes roezlii* | MN496076 |
|  |  | *Ribes glaciale* | NC062156 |
|  |  | *Ribes odoratum* | MT081309 |
|  |  | *Ribes fasciculatum* var. *chinense* | MH191388 |
| Saxifragaceae | *Saxifraga* | *Saxifraga stolonifera* | MN496079 |
|  | *Chrysosplenium* | *Chrysosplenium ramosum* | MK973002 |
|  | *Astilbe* | *Astilbe chinensis* | MK990829 |
|  | *Heuchera* | *Heuchera micrantha* | OL489769 |
|  | *Rodgersia* | *Rodgersia aesculifolia* | NC057229 |
|  | *Oresitrophe* | *Oresitrophe rupifraga* | MF774190 |
|  | *Bergenia* | *Bergenia purpurascens* | OK012000 |
| Iteaceae | *Itea* | *Itea chinensis* | NC037884 |
| Cercidiphyllaceae | *Cercidiphyllum* | *Cercidiphyllum magnificum* | NC046692 |
| Hamamelidaceae | *Semiliquidambar* | *Semiliquidambar cathayensis* | MN410884 |
| Altingiaceae | *Liquidambar* | *Liquidambar formosana* | MN623380 |
|  |  | *Liquidambar chinensis* | NC047288 |
| Paeoniaceae | *Paeonia* | *Paeonia suffruticosa* | JQ952559 |
|  |  | *Paeonia brownii* | JQ952560 |
|  |  | *Paeonia obovata* | JQ952561 |
| Crassulaceae | *Sedum* | *Sedum oryzifolium* | NC027837 |
|  | *Hylotelephium* | *Hylotelephium ewersii* | MN794014 |
| Haloragaceae | *Myriophyllum* | *Myriophyllum spicatum* | MK250869 |
| Penthoraceae | *Penthorum* | *Penthorum chinense* | NC023086 |
| Celastraceae | *Parnassia* | *Parnassia wightiana* | NC061947 |
| Rosaceae | *Rosa* | *Rosa laevigata* var. *leiocarpa* | NC047418 |
| Rhamnaceae | *Rhamnus* | *Rhamnus globosa* | ON009012 |


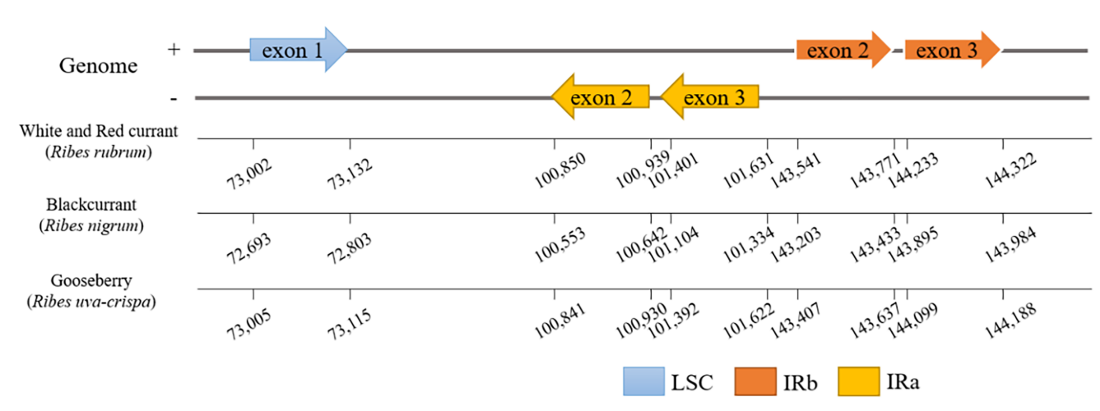


**Figure S1**. Trans-splicing structure of *rps12* in the four *Ribes* cp genomes

Distance in the figure is not to scale.


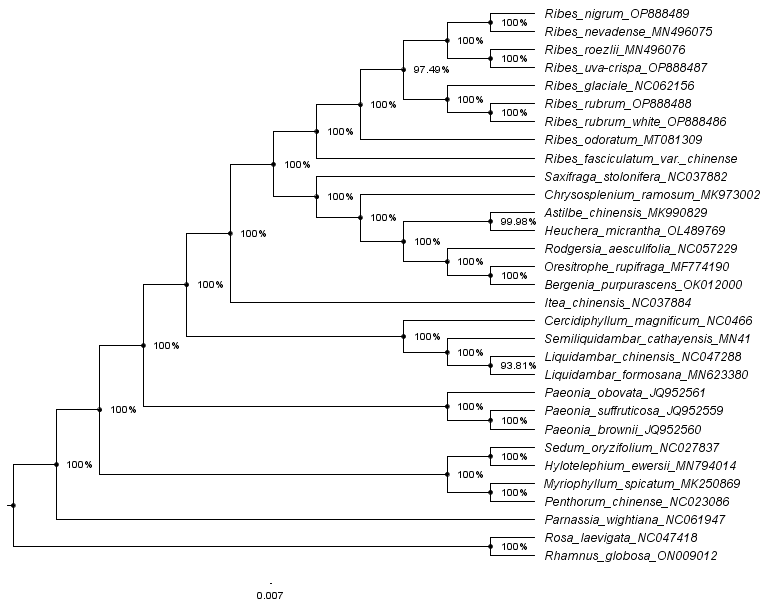


**Figure S2**. Phylogenetic tree inferred using Bayesian Inference

The phylogenetic tree was constructed based on CDS sequence of the 31 cp genomes using BEAST 2.7.5. Posterior probabilities are shown on the right of the nodes.
